# Housing mice in the individually ventilated or open cages—Does it matter for behavioral phenotype?

**DOI:** 10.1111/gbb.12564

**Published:** 2019-03-28

**Authors:** Johanna Åhlgren, Vootele Voikar

**Affiliations:** ^1^ Neuroscience Center, Helsinki Institute of Life Science University of Helsinki Helsinki Finland; ^2^ Laboratory Animal Center, Helsinki Institute of Life Science University of Helsinki Helsinki Finland

**Keywords:** mice, housing, environment, phenotyping, behavior, IVC, sex, inbred, reproducibility, species‐specific

## Abstract

Individually ventilated caging (IVC) systems for rodents are increasingly common in laboratory animal facilities. However, the impact of such substantial change in housing conditions on animal physiology and behavior is still debated. Most importantly, there arise the questions regarding reproducibility and comparison of previous or new phenotypes between the IVC and open cages. The present study was set up for detailed and systematic comparison of behavioral phenotypes in male and female mice of three widely used inbred strains (C57BL/6JRccHsd, DBA/2JRccHsd, 129S2/SvHSd) after being kept in two housing environments (IVC and open cages) for 6 weeks (since 4 weeks of age) before behavioral testing. The tests addressed exploratory, anxiety‐like and stress‐related behavior (light‐dark box, open field, forced swim test, stress‐induced hyperthermia), social approach and species‐specific behavior (nest building, marble burying). In all tests, large and expected strain differences were found. Somewhat surprisingly, the most striking effect of environment was found for basal body temperature and weight loss after one night of single housing in respective cages. In addition, the performance in light‐dark box and open field was affected by environment. Several parameters in different tests showed significant interaction between housing and genetic background. In summary, the IVC housing did not invalidate the well‐known differences between the mouse strains which have been established by previous studies. However, within the strains the results can be influenced by sex and housing system depending on the behavioral tasks applied. The bottom‐line is that the environmental conditions should be described explicitly in all publications.

## Graphical abstract

The individually ventilated caging housing did not invalidate the well‐known differences between the mouse strains established by previous studies. However, within the strains the results can be influenced by sex and housing system depending on the behavioral tasks applied.

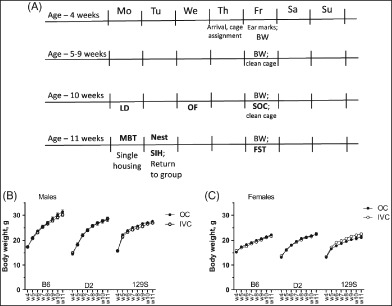

## 1 INTRODUCTION

Phenotype is a result of interaction between the organism's genotype and environment. Mouse has evolved as a species of choice for basic biomedical research, especially for investigating the role of genetic factors in physiology and pathology. International consortia are aiming at finding the functional role of every single gene in mouse genome, and ultimate goal of all animal research is to improve cure for human disorders.^1^

However, at the time when scientific literature using mouse as a research subject is increasing exponentially, serious concerns have been expressed regarding the validity of animal studies, with emphasis on reproducibility and external validity of the research findings.^2^, ^3^ Among many factors contributing to these problems, environment in its broad sense is certainly one of the major issues. It is too often when the differences between the results by different laboratories are referred to be caused by environmental or procedural differences. As a matter of fact, this is not necessarily an unexpected or negative outcome.^4^ There have been several attempts to standardize the research methods and protocols.^5^ However, it appears that extreme standardization can yield the results idiosyncratic to particular laboratory, and therefore, standardization may not be the best way for improving external validity of animal models.^6^, ^7^ Nevertheless, complete reporting of the details of experimental design is an essential and mandatory part of scientific publications, although often neglected.^8^, ^9^ Despite all efforts made so far for improving the standards for reporting, the impact on the quality of reporting has been limited.^10^

The full life history of the animals can be considered even more important determinant of the phenotype in comparison to the particular test protocol. In this respect, the environment of animal cage (and animal facilities in general) has been underestimated. Certainly, some aspects of housing conditions have been highlighted by earlier research^11^ and in the current legislation regulating animal welfare—for example, social housing and appropriate enrichment.^12^ Already these developments have caused some concerns in community.^13^ In addition, during last 10‐15 years most of the newly built or renovated animal facilities are equipped with the individually ventilated caging (IVC) systems. The IVC has been suggested to be useful for controlling the spread of infections between animals, to maintain immune and health conditions at required levels, but also beneficial for staff by reducing air pollution and allergens.^14^, ^15^ However, from the animal viewpoint this can be considered as a major difference and re‐validation of the established phenotypes is warranted. Indeed, there are several reports published where behavioral phenotypes have been affected by moving the animals from the open cages (OCs) to IVC (see Table 1).

**Table 1:** References to the previous studies assessing the effect of IVC housing on behavioral phenotype of mice

References	Animals and housing	Main findings
Burman et al^26^	C57BL/6J, Balb/c; Females; age 6‐7 weeks at start (obtained from Charles River); group‐housed; behavioral testing after 7 weeks; two IVC systems (SealSafe Plus[Tecniplast] and Allentown)	Two IVC systems (air delivery at cage cover or animal level)—increased anxiety‐like behavior in elevated plus‐maze in mice from cages with air delivery at animal level; Results suggest that different IVC housing systems can influence mouse behavior in different ways
Kallnik et al^34^	C3HeB/FeJ, C57BL/6J; Males; born in IVC (VentiRacks, BioZone, Margate, UK) single housed from weaning, either in IVC or open cages; behavioral testing at the age of 9‐14 weeks	IVC housing reduced activity and enhanced anxiety‐related behavior in both strains, whereas grooming latency was reduced in B6J only. IVC housing increased Acoustic Startle Response in C3H but not in B6J mice. IVC housing can affect behavioral performance and can modulate behavioral parameters in a general and a strain‐specific manner
Logge et al^48^	C57BL/6JArc (Australian BioResources); Male and Female; group‐housed; born and raised in IVC (Airlaw) or OPEN; at 5 months of age transferred to testing lab and housed in OPEN	IVC had anxiety‐like effects in the elevated plus maze, which were more pronounced in female mice whereas cognition and locomotion of all test mice were not modified by IVC housing. Mice raised in IVC cage systems were socially more active than mice of filter‐top systems. Differences between the housing conditions of breeding facilities and test facilities must carefully be considered
Logge et al^49^	Neuregulin 1 mutant mice (+ wild type controls) on C57BL/6JArc background; Male and Female; group‐housed; born and raised in IVC (Airlaw) or OPEN; at 5‐6 months of age transferred to testing lab and housed in OPEN	IVCs diminished the schizophrenia‐relevant pre‐pulse inhibition deficit of Nrg1 mutant males. Furthermore, IVC housing had a sex‐dependent moderate effect on the locomotive phenotype of Nrg1 mice across test paradigms. Behavioral effects of IVC housing were less prominent in female mice
Mineur & Crusio^25^	BALB/cJ, C57BL/6J, DBA/2J; males and females, group‐housed, born in open cages, after weaning transferred to open or IVC (Allentown Caging Equipment); testing at the age of 3 months	Results show robust effects of IVC in multiple behavioral tests (assessing anxiety, exploration, learning) with the direction of the effect strongly dependent on strain and sex
Pasquarelli et al^37^	C57BL/6JRj male mice, approx. 20 g in the beginning (!), 15 days of habituation in OPEN cages, followed by transfer to IVC (Greenline IVC SealSafe PLUS, Tecniplast) or OPEN in groups or isolated; behavioral testing carried out during 40 days after transfer	Data indicate a crucial influence of a change in housing conditions on several mouse phenotype parameters, for example, Elevated plus maze test showed that a change to IVC single and social housing as well as single standard housing produced anxiety‐related behavior when compared to maintenance in social standard housing
Polissidis et al^42^	C57BL/6J; males, group‐housed, assigned to different caging (open; IVC with positive pressure; MFVC—motor free ventilated cages with negative pressure) at age of 8 weeks, testing at the age of 12 weeks	Although there were no differences in the open field test, the results from the elevated plus maze showed that animals housed in the MFVCs exhibited increased exploratory and less anxiety‐like behavior. It is concluded that the different caging systems may have an impact on the outcome of behavioral tests used to assess exploratory and anxiety like behavior in mice

Abbreviations: IVC, individually ventilated caging.

Therefore, the goal of the present study was a systematic evaluation of basic behavioral phenotypes in male and female mice of three commonly used inbred strains (C57BL/6JRccHsd, DBA/2JRccHsd, 129S2/SvHsd) housed in IVC or OCs. For comprehensive characterization, we designed a test battery with the focus on exploratory activity, social, species‐specific and stress‐related behavior. This approach was based on suggestions and recommendations by earlier studies addressing the concept of test batteries and the possible effect of repeated testing.^16^, ^17^, ^18^, ^19^

## 2 MATERIALS AND METHODS

### 2.1 Ethics statement, animals and environmental conditions

The animal experiments were performed according to the European Union legislation harmonized with Finnish legislation and have been approved by the National Animal Experiment Board of Finland (ESAVI/10165/04.10.07/2016).

All animals were ordered from Envigo (Horst, The Netherlands) and arrived in Helsinki at the age of 4 weeks. Altogether, 24 male and 24 female mice from each of the following inbred strains were ordered for this study (144 in total): C57BL/6JRccHsd (B6) mice, DBA/2JRccHsd (D2) and 129S2/SvHsd (129). The number of animals was decided according to the previous experience and suggestions in literature for detecting the main effects in factorial design (strain, sex and housing condition as main factors).^16^, ^20^ The mice were delivered in four batches with interval of 2 weeks (first and third batch—males, second and fourth batch—females; 12 animals per strain in each batch), and were randomly allocated (random number calculator, available at https://www.graphpad.com/quickcalcs/) to the groups of three animals per cage between two housing conditions:

1. individually ventilated plastic cages in animal room (Mouse IVC Green Line—overall dimensions 391 × 199 × 160 mm, floor area 501 cm^2^; Tecniplast, Buguggiate,Italy) with half of the cage covered by wire bar food hopper. Air inlet and outlet valves are located in the cage lid, on top of the cage and rate of air change was set at 75 times per hour with air speed at animal level max. 0.05 m/s;
2. open‐top, type II cages (dimensions 267 × 207 × 140 mm—floor area cm^2^ 370; Tecniplast) covered by wire bar lid and kept in a cabinet (Scantainer) ventilated with room air in animal room.

On the next day after arrival, the mice were ear punched for identification and the body weight was measured. In both housing conditions, enrichment was provided by bedding (aspen chips 5 × 5 × 1 mm, 4HP, Tapvei, Kiili, Estonia), nesting material (aspen strips, PM90L, Tapvei and a pressed cotton square, Nestlets; Ancare, Bellmore, New York) and aspen brick (100 × 20 × 20 mm, Tapvei). Food (Global Diet 2916C, pellet 12 mm, Envigo, Horst, The Netherlands) and water (filtered and ultraviolet‐irradiated) was available ad libitum. Room temperature was 22 ± 2°C and relative humidity 50 ± 15%. The lights were on between 6:00 and 18:00. The cages were cleaned once per week, suitable nesting material was transferred to the new cage in order to reduce aggressiveness and facilitate adaptation.^21^, ^22^ Animals were weighed before moving to the clean cage.

One C57BL/6J male mouse from the first batch (OC) was found dead next morning after arrival, and one C57BL/6J male mouse from the second batch (OC) was removed at the age of 9 weeks because of aggressive behavior towards the cage‐mates. Accordingly, the number of mice in each sub‐group (per strain, sex and housing condition) was twelve, except for C57BL/6J males in OCs (n = 10).

### 2.2 Behavioral tests

**Figure 1:**
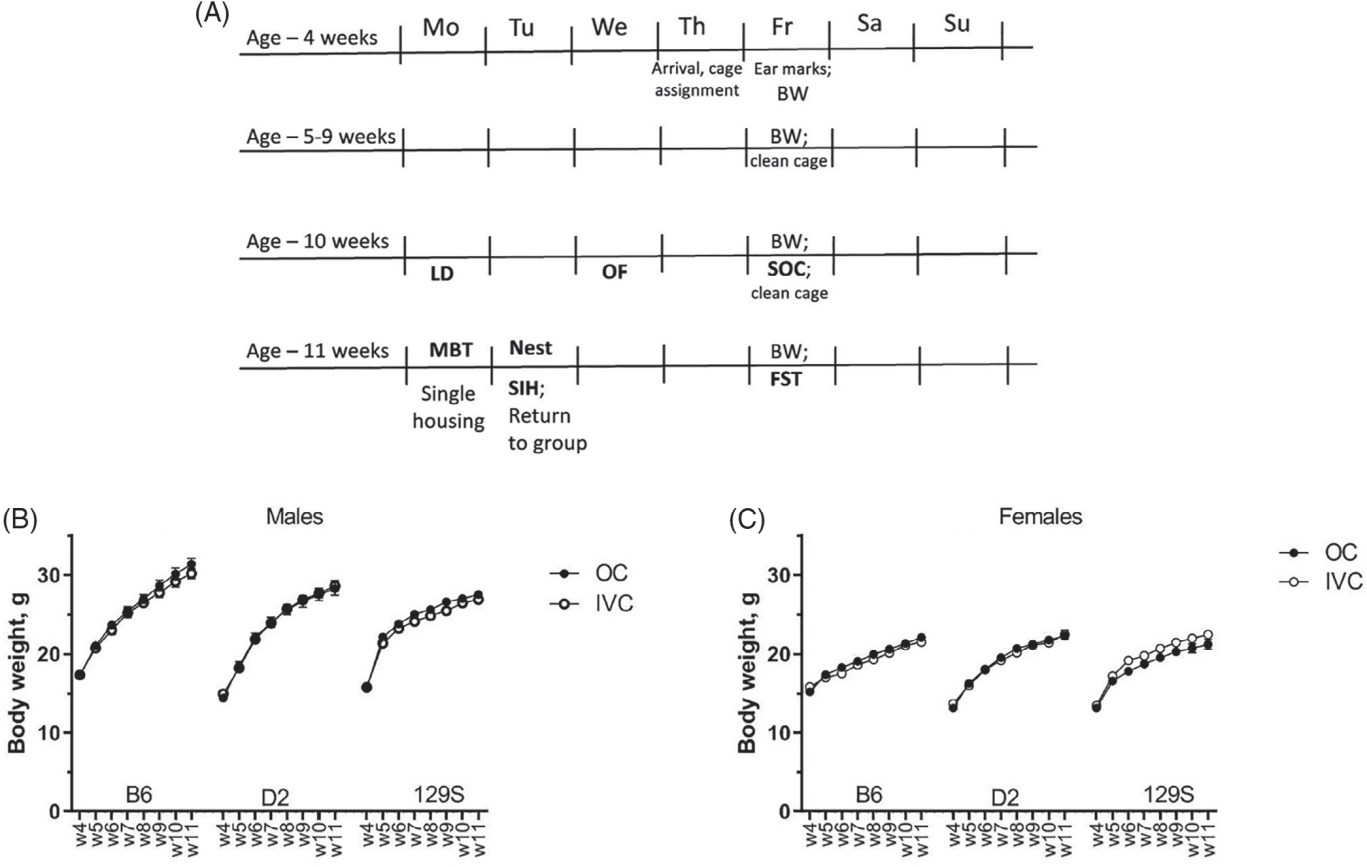
A, Timeline of the experiment. The mice arrived at the age of 4 weeks and randomly assigned to either IVC or open cages (OC). After 5 weeks of adaptation (age of mice 5‐9 weeks), the behavioral test battery was carried out during 2 weeks (age of mice 10‐11 weeks): FST, forced swim test; LD, light‐dark test; MBT, marble burying test; Nest, nest building; OF, open field; SIH, stress‐induced hyperthermia; SOC, social approach. B, Body weight of male mice during the observation period (age 4‐11 weeks). C, Body weight of female mice during the observation period (age 4‐11 weeks). Data are expressed as mean ± SEM; N = 10 B6 males in open field (OC); N = 12 for all other groups

Testing started when the animals were 10 weeks old (ie, after 6 weeks of adaptation) and a battery of behavioral tests was carried out during 2 weeks in the order described below. According to arrival, testing was also carried out in four blocks by two experimenters and sequence of animals for separate tests was randomized. For all tests the animals were moved to the testing room at least 30 minutes before the start and testing order was randomized each time. Experiments were carried out during the light period (between 8:00 and 16:00) in the following order (Figure 1A)—light‐dark (LD) box (day 1); open field (OF, day 3); social exploration (SOC, day 5); marble burying test (MBT, day 8); Nest (day 8‐9); stress‐induced hyperthermia (SIH, day 9); forced swimming test (FST, day 12).

#### 2.2.1 LD box

The test was carried out in the square open field arena (30 × 30 × 20 cm; Med Associates, St. Albans, Vermont) equipped with infrared light sensors detecting horizontal and vertical activity. The dark insert (nontransparent for visible light, light intensity <5 lx) was used to divide the arena into two halves, an opening (a door with a width of 5.5 cm and height of 7 cm) in the wall of the insert allowed animal's free movement from one compartment to another. Illumination in the center of the light compartment was approximately 550 lx. Animal was placed in the dark compartment and allowed to explore the arena for 10 minutes. Latency to enter the light side, distance travelled, number of rearings, and time spent in different compartments were recorded by the program (Activity Monitor, version 5.8). The number of fecal boli was counted by experimenter after the end of the trial.

#### 2.2.2 Open field

The same arena as for the LD test was used without the dark insert, illumination of the arena was approximately 150 lx. Animals were released in the corner of the arena and monitored for 20 minutes. For analysis, the arena was divided into the center and periphery, the peripheral zone defined as a 6 cm wide corridor along the wall.

#### 2.2.3 Social exploration

Large cage (dimensions 480 × 375 × 210 mm) contained two transparent and perforated cylinders (diameter 9 cm, height 15 cm), placed at the center of opposite short walls (distance between the cylinders 30 cm). One of the cylinders contained a social stimulus (unfamiliar age‐ and sex‐matched NMRI mouse [Envigo], kept in group‐housing and previously adapted to confinement in the cylinder). The test was performed under reduced light conditions (approximately 30 lx). Mice were released in the center of arena and behavior was recorded by Ethovision XT 10.0 during 10 minutes. Total distance travelled, time spent close to the cylinders (5 cm zone surrounding the cylinder) and average proximity to the cylinders were measured.

#### 2.2.4 Marble burying test

Test was performed in the IVC cage with a thick layer (4‐5 cm) of dampened bedding. The cage was covered by a perforated transparent polyvinyl chloride‐lid. Twenty glass marbles were placed on top of the bedding (arranged in 4 × 5) and mice were allowed to explore the cage and interact with the marbles for 30 minutes. Thereafter, the mice were returned to the home cage, the test cages were labeled and the marbles were counted into three categories (covered fully [hidden], less than 50% or more than 50% of the marble visible) by experimenter blinded regarding the identity of the subjects.

#### 2.2.5 Nest construction

In the afternoon (2 hours before dark onset) the mice were individually moved to the new cages (the same type as for group housing), with food and water available ad libitum and one intact Nestlet (5 cm square, approximately 2.5 g) as nesting material. Thus, the nesting material was familiar for the animals. Quality of the nest was assessed and scored next morning (2 hours after beginning of the light period, that is, 16 hours after beginning the trial) according to 5‐point scale.^23^

#### 2.2.6 Stress‐induced hyperthermia

After visual inspection and assessment of the nest, the mice were removed from the cage and rectal temperature was measured. Then the body weight was measured and animal was returned to the cage, 10 minutes later measurement of rectal temperature was repeated. The difference between two measurements is defined as a stress‐induced hyperthermia. Thereafter, the mice were returned to their original home cages in groups of three.

#### 2.2.7 Forced swimming test

The mouse was placed for 6 minutes in the glass cylinder (Ø 18 cm, height 25 cm) filled with water at 23 ± 1°C to the height of 15 cm. The time of immobility (passive floating, when the animal was motionless or doing only slight movements with tail or one hind limb, whereas the animal was judged to be active when struggling, climbing or swimming using all four paws) was measured in 2‐minute intervals. Behavior was video recorded and immobility episodes were detected by Ethovision XT 10.0 (Noldus) software.

#### 2.2.8 Statistics

All statistical analyses were conducted using IBM SPSS Statistics 25. Data from behavioral analysis were subjected to Univariate Analysis of Variance or Analysis of Variance of repeated measures (General Linear Model); OF and LD in 5‐minute bins, SOC in 1‐minute bins and FST in 2‐minute bins with a Bonferroni correction for a multiple comparisons when appropriate. Environment, strain and sex were applied as between‐subject factors (fixed factors) for calculating the main effects and respective interactions between the factors (the results for main outcome variables are shown in Table 2). In case of significant interactions, data were subjected to analysis of variance split by strain and by sex with remaining interactions. Significance level was set to *P* < 0.05. Data are presented as means ± SEM.

**Table 2:** Summary of ANOVA results for main variables, significant findings are highlighted

Parameter	Housing	Strain	Sex	Housing^*^Strain	Housing^*^Sex	Strain^*^Sex	Housing^*^Strain^*^Sex
LD: Latency to light, s	*F*_(1,130)_ = 0.72, *P* = 0.40	*F*_(2,130)_ = 12.80, *P* < 0.01	*F*_(1,130)_ = 0.31, *P* = 0.58	*F*_(2,130)_ = 0.08, *P* = 0.93	*F*_(1,130)_ = 0.34, *P* = 0.56	*F*_(2,130)_ = 0.62, *P* = 0.54	*F*_(2,130)_ = 0.80, *P* = 0.45
LD: Total distance, cm	*F*_(1,130)_ = 1.27, *P* = 0.26	*F*_(2,130)_ = 110.52, *P* < 0.01	*F*_(1,130)_ = 0.49, *P* = 0.48	*F*_(2,130)_ = 0.05, *P* = 0.95	*F*_(1,130)_ = 0.008, *P* = 0.93	*F*_(2,130)_ = 4.03, *P* = 0.02	*F*_(2,130)_ = 0.51, *P* = 0.60
LD: Distance light%	*F*_(1,130)_ = 5.67, *P* = 0.02	*F*_(2,130)_ = 17.22, *P* < 0.01	*F*_(1,130)_ = 4.64, *P* = 0.03	*F*_(2,130)_ = 1.22, *P* = 0.30	*F*_(1,130)_ = 1.70, *P* = 0.20	*F*_(2,130)_ = 0.68, *P* = 0.51	*F*_(2,130)_ = 2.10, *P* = 0.13
LD: Time light%	*F*_(1,130)_ = 6.73, *P* = 0.01	*F*_(2,130)_ = 20.94, *P* < 0.01	*F*_(1,130)_ = 2.46, *P* = 0.12	*F*_(2,130)_ = 2.08, *P* = 0.13	*F*_(1,130)_ = 1.56, *P* = 0.21	*F*_(2,130)_ = 0.05, *P* = 0.95	*F*_(2,130)_ = 0.87, *P* = 0.42
LD: Total rearings, nr	*F*_(1,130)_ = 0.58, *P* = 0.45	*F*_(2,130)_ = 133.28, *P* < 0.01	*F*_(1,130)_ = 9.63, *P* < 0.01	*F*_(2,130)_ = 1.94, *P* = 0.15	*F*_(1,130)_ = 0.07, *P* = 0.79	*F*_(2,130)_ = 1.43, *P* = 0.24	*F*_(2,130)_ = 0.53, *P* = 0.59
LD: Rearings in light%	*F*_(1,130)_ = 6.82, *P* = 0.01	*F*_(2,130)_ = 14.50, *P* < 0.01	*F*_(1,130)_ = 3.59, *P* = 0.06	*F*_(2,130)_ = 0.71, *P* = 0.49	*F*_(1,130)_ = 0.001, *P* = 0.99	*F*_(2,130)_ = 1.74, *P* = 0.18	*F*_(2,130)_ = 0.06, *P* = 0.95
OF: Total distance, cm	*F*_(1,130)_ = 0.09, *P* = 0.76	*F*_(2,130)_ = 227.76, *P* < 0.01	*F*_(1,130)_ = 13.55, *P* < 0.01	*F*_(2,130)_ = 6.22, *P* < 0.01	*F*_(1,130)_ = 1.13, *P* = 0.29	*F*_(2,130)_ = 10.69, *P* < 0.01	*F*_(2,130)_ = 3.70, *P* = 0.03
OF: Distance center%	*F*_(1,130)_ = 0.002, *P* = 0.96	*F*_(2,130)_ = 141.16, *P* < 0.01	*F*_(1,130)_ = 0.006, *P* = 0.94	*F*_(2,130)_ = 8.73, *P* < 0.01	*F*_(1,130)_ = 0.46, *P* = 0.50	*F*_(2,130)_ = 4.42, *P* = 0.01	*F*_(2,130)_ = 1.65, *P* = 0.20
OF: Time center%	*F*_(1,130)_ = 3.99, *P* = 0.05	*F*_(2,130)_ = 222.85, *P* < 0.01	*F*_(1,130)_ = 0.05, *P* = 0.83	*F*_(2,130)_ = 13.86, *P* < 0.01	*F*_(1,130)_ = 0.08, *P* = 0.77	*F*_(2,130)_ = 1.26, *P* = 0.29	*F*_(2,130)_ = 0.87, *P* = 0.42
OF: Total rearings, nr	*F*_(1,130)_ = 0.73, *P* = 0.39	*F*_(2,130)_ = 309.37, *P* < 0.01	*F*_(1,130)_ = 0.08, *P* = 0.78	*F*_(2,130)_ = 2.00, *P* = 0.14	*F*_(1,130)_ = 0.05, *P* = 0.83	*F*_(2,130)_ = 0.26, *P* = 0.78	*F*_(2,130)_ = 0.15, *P* = 0.86
OF: Rearings in center%	*F*_(1,130)_ = 2.37, *P* = 0.13	*F*_(2,130)_ = 93.81, *P* < 0.01	*F*_(1,130)_ = 5.23, *P* = 0.02	*F*_(2,130)_ = 5.31, *P* < 0.01	*F*_(1,130)_ = 0.88, *P* = 0.35	*F*_(2,130)_ = 5.22, *P* < 0.01	*F*_(2,130)_ = 0.89, *P* = 0.41
SOC: Total distance, cm	*F*_(1,130)_ = 0.008, *P* = 0.93	*F*_(2,130)_ = 202.46, *P* < 0.01	*F*_(1,130)_ = 11.45, *P* < 0.01	*F*_(2,130)_ = 2.23, *P* = 0.11	*F*_(1,130)_ = 3.00, *P* = 0.09	*F*_(2,130)_ = 5.04, *P* < 0.01	*F*_(2,130)_ = 0.37, *P* = 0.69
SOC: Time interact. Zone	*F*_(1,130)_ = 0.11, *P* = 0.75	*F*_(2,130)_ = 9.68, *P* < 0.01	*F*_(1,130)_ = 0.92, *P* = 0.34	*F*_(2,130)_ = 0.52, *P* = 0.60	*F*_(1,130)_ = 1.96, *P* = 0.16	*F*_(2,130)_ = 0.28, *P* = 0.76	*F*_(2,130)_ = 1.29, *P* = 0.28
MBT: Fully buried, nr	*F*_(1,130)_ = 3.33, *P* = 0.07	*F*_(2,130)_ = 70.81, *P* < 0.01	*F*_(1,130)_ = 4.00, *P* = 0.05	*F*_(2,130)_ = 0.12, *P* = 0.89	*F*_(1,130)_ = 3.52, *P* = 0.06	*F*_(2,130)_ = 1.49, *P* = 0.23	*F*_(2,130)_ = 3.29, *P* = 0.04
Nest score	*F*_(1,130)_ = 0.80, *P* = 0.37	*F*_(2,130)_ = 55.04, *P* < 0.01	*F*_(1,130)_ = 2.90, *P* = 0.09	*F*_(2,130)_ = 2.63, *P* = 0.76	*F*_(1,130)_ = 3.48, *P* = 0.06	*F*_(2,130)_ = 11.53, *P* < 0.01	*F*_(2,130)_ = 0.56, *P* = 0.57
Body weight change, %	*F*_(1,130)_ = 11.72, *P* < 0.01	*F*_(2,130)_ = 13.30, *P* < 0.01	*F*_(1,130)_ = 21.76, *P* < 0.01	*F*_(2,130)_ = 0.74, *P* = 0.48	*F*_(1,130)_ = 1.74, *P* = 0.19	*F*_(2,130)_ = 3.72, *P* = 0.03	*F*_(2,130)_ = 0.22, *P* = 0.80
Basal temperature	*F*_(1,130)_ = 19.25, *P* < 0.01	*F*_(2,130)_ = 0.05, *P* = 0.95	*F*_(1,130)_ = 31.86, *P* < 0.01	*F*_(2,130)_ = 1.05, *P* = 0.35	*F*_(1,130)_ = 0.10, *P* = 0.75	*F*_(2,130)_ = 0.54, *P* = 0.59	*F*_(2,130)_ = 0.68, *P* = 0.51
SIH: temp. change	*F*_(1,130)_ = 2.86, *P* = 0.09	*F*_(2,130)_ = 4.95, *P* < 0.01	*F*_(1,130)_ = 35.66, *P* < 0.01	*F*_(2,130)_ = 3.55, *P* = 0.03	*F*_(1,130)_ = 1.71, *P* = 0.19	*F*_(2,130)_ = 1.09, *P* = 0.34	*F*_(2,130)_ = 2.16, *P* = 0.12
FST: immobility, s	*F*_(1,130)_ = 0.004, *P* = 0.95	*F*_(2,130)_ = 18.92, *P* < 0.01	*F*_(1,130)_ = 5.00, *P* = 0.03	*F*_(2,130)_ = 2.20, *P* = 0.11	*F*_(1,130)_ = 0.11, *P* = 0.75	*F*_(2,130)_ = 1.52, *P* = 0.22	*F*_(2,130)_ = 0.66, *P* = 0.52

Abbreviations: FST, orced swim test; LD, light‐dark box; MBT, marble burying test; OF, open field; SIH, stress‐induced hyperthermia; SOC, social approach.

## 3 RESULTS

#### 3.1.1. Body weight

Body weight of the animals was recorded weekly during the age of 4 weeks to the age of 11 weeks (Figure 1B,C). There was no difference between animals housed in IVC or in OCs (males *F*_1,64_ = 0.94, *P* = 0.34; females *F*_1,66_ = 0.34, *P* = 0.56). Significant strain difference in body weight was established in male (*F*_2,64_ = 8.1, *P* < 0.001, B6 > D2,129) but not in female mice (*F*_2,66_ = 2.06, *P* = 0.14). However, the interaction between housing environment and strain was not significant, neither in males nor females.

#### 3.1.2. Light‐dark box

**Figure 2:**
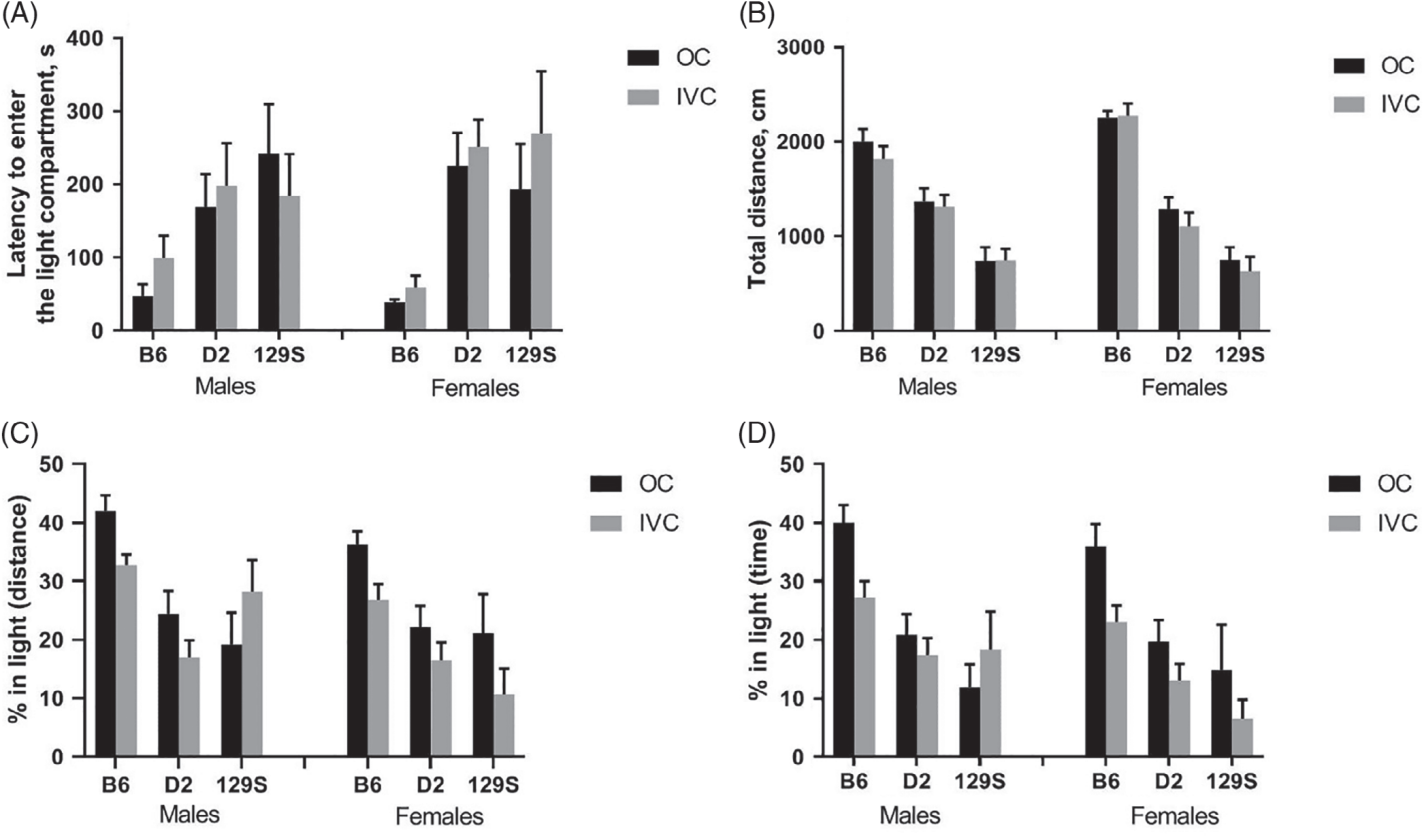
Results of anxiety‐like and exploratory behavior in the light‐dark box. A, Latency to the first entry from dark to light compartment. B, Total distance in travelled during 10 minutes of testing. C, Percentage of the distance travelled in the light compartment. D, Percentage of time spent in the light compartment during 10 minutes test. Data are expressed as mean ± SEM; N = 10 B6 males in open field (OC); N = 12 for all other groups

The latency to enter the light compartment (Figure 2A) was not affected by housing conditions (*F*_1,130_ = 0.72, *P* = 0.4), whereas it was significantly shorter in B6 mice compared to other strains (*F*_2,130_ = 12.8, *P* < 0.001). The general activity (total distance travelled during 10 minutes, Figure 2B) was not affected by housing conditions (*F*_1,130_ = 1.27, *P* = 0.26), whereas strains differed significantly with B6 mice being the most and 129 mice the least active (*F*_2,130_ = 110.52, *P* < 0.0001). The proportion of distance travelled and time spent in the light compartment (Figure 2C,D) was significantly enhanced in animals housed in OCs (*F*_1,130_ = 5.67, *P* = 0.02 and *F*_1,130_ = 6.73, *P* = 0.01, respectively). For both parameters, the B6 mice displayed higher value than D2 and 129 mice (strain difference for distance *F*_2,130_ = 17.22, *P* < 0.0001 and for time *F*_2,130_ = 20.94, *P* < 0.0001).

#### 3.1.3. Open field

**Figure 3:**
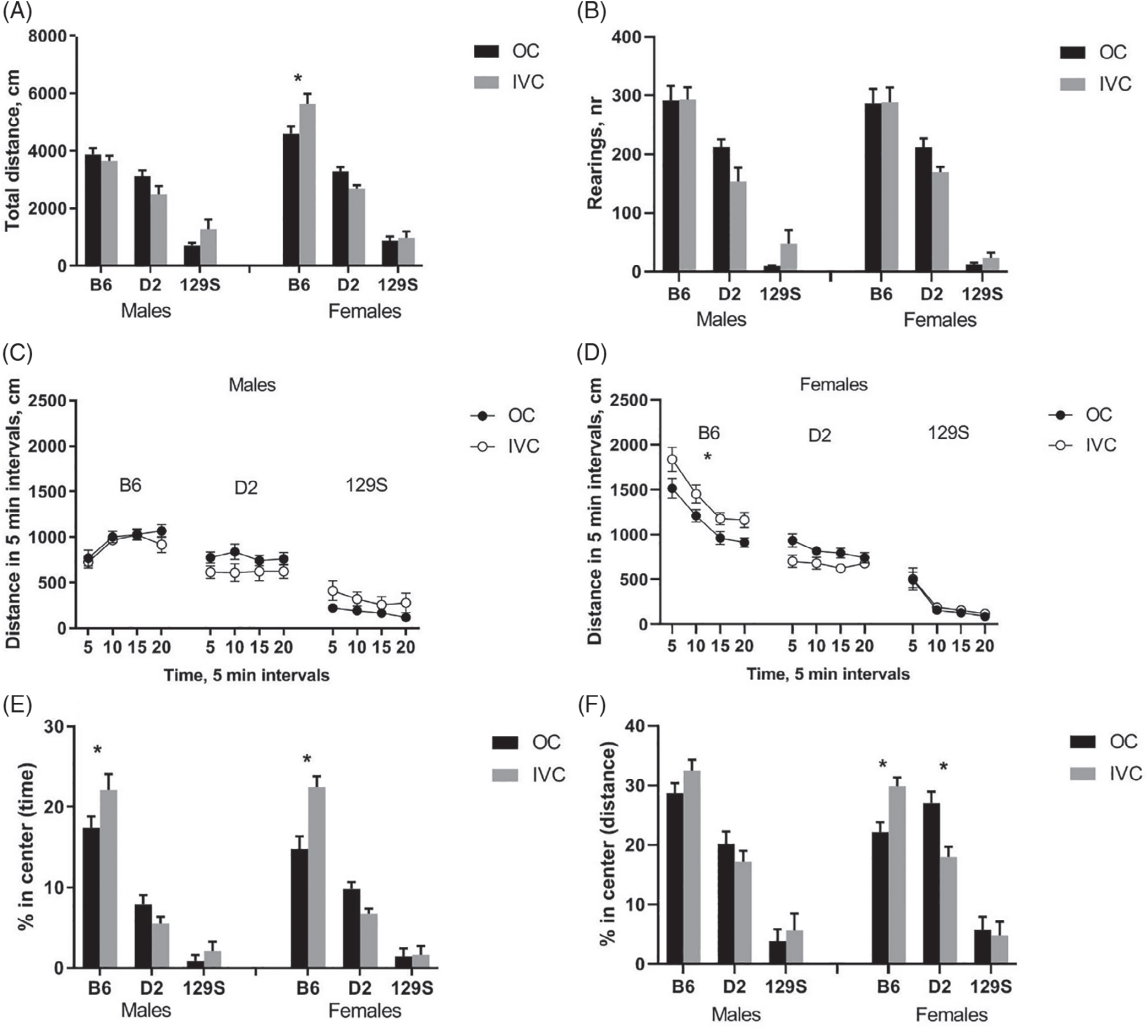
Results of exploratory behavior in the open field arena. A, Total distance travelled during 20 minutes of testing. B, Number of rearings during test. C, Distance travelled in 5‐minute intervals (males). D, Distance travelled in 5‐minute intervals (females). E, Percentage of time spent in the center of open field arena during 20 minutes of testing. F, Percentage of distance in the center of open field arena during 20 minutes of testing. Data are expressed as mean ± SEM; N = 10 B6 males in open field (OC); N = 12 for all other groups; *P < 0.05 Bonferroni post‐hoc comparison

Similar to LD‐test, there was no overall difference in total activity (distance travelled, Figure 3A,C,D) related to housing environment (*F*_1,130_ = 0.09, *P* = 0.76), whereas the strains differed remarkably (*F*_2,130_ = 227.76, *P* < 0.0001, B6 > D2 > 129). Moreover, the female mice showed increased activity compared to the males (*F*_1,130_ = 13.55, *P* = 0.0003). These findings in main effects were further complicated by several significant interactions (environment by strain *F*_2,130_ = 6.22, *P* = 0.0026; strain by sex *F*_2,130_ = 10.69, *P* < 0.0001; environment by strain by sex *F*_2,130_ = 3.70, *P* = 0.0274) indicating that D2 mice of both sexes in IVC cages tended to move less than D2 mice in OCs, and B6 female and 129 male mice in IVC tended to move more compared to respective groups in OCs. Another parameter reflecting locomotor and exploratory activity, the number of rearings (Figure 3B), differed significantly between the strains (*F*_2,130_ = 309.37, *P* < 0.0001, B6 > D2 > 129) but not between the housing conditions (*F*_1,130_ = 0.73, *P* = 0.39). With regard to anxiety‐related parameters, the proportion of distance and time in the central area of the OF (Figure 3E,F) were not substantially affected by housing conditions (*F*_1,130_ = 0.002, *P* = 0.96 and *F*_1,130_ = 3.99, *P* = 0.05), although significant interaction of environment and strain for both parameters (*F*_2,130_ = 8.73, *P* = 0.0003 and *F*_2,130_ = 13.86, *P* < 0.0001) indicated a difference between B6 and D2 mice (B6 mice from OCs displayed avoidance to center area compared to B6 from IVC, whereas D2 mice displayed an opposite pattern).

#### 3.1.4. Social approach

**Figure 4:**
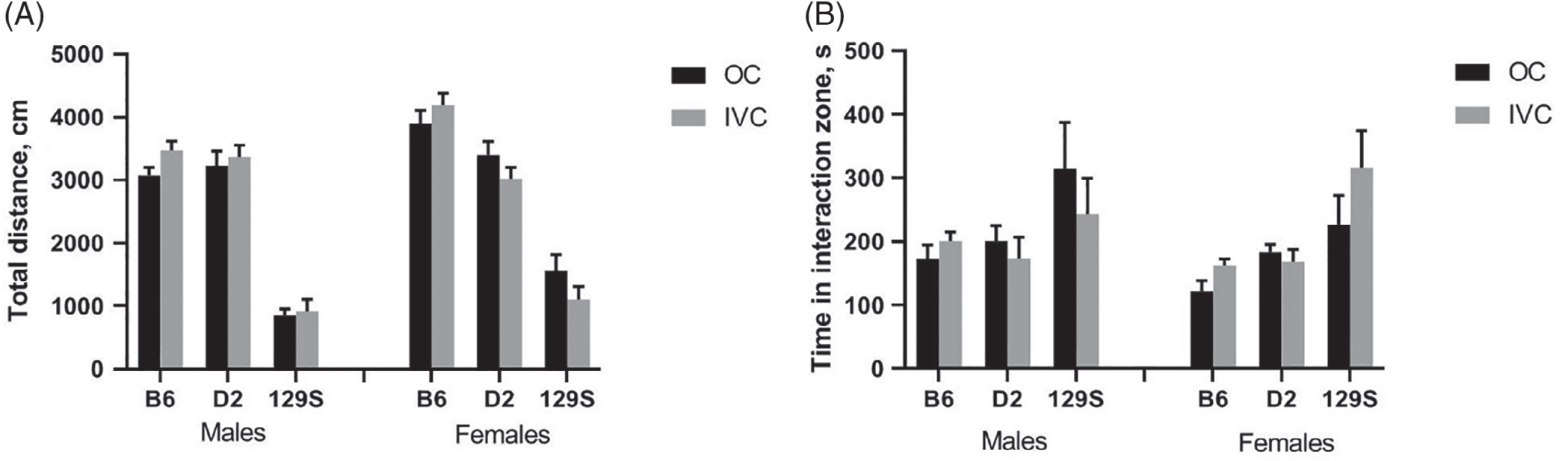
Social approach. A, distance travelled during 10 minutes of testing. B, Time in social interaction zone (surrounding perforated cylinder with stimulus mouse). Data are expressed as mean ± SEM; N = 10 B6 males in open field (OC); N = 12 for all other groups

Total activity during 10 minutes of testing (Figure 4A) was not affected by housing (*F*_1,130_ = 0.01, *P* = 0.93) whereas strains differed markedly (*F*_2,130_ = 202.5, *P* < 0.0001, B6 > D2 > 129). The time spent close to the compartment containing unfamiliar stimulus mouse (Figure 4B) was neither affected by housing conditions (*F*_1,130_ = 0.11, *P* = 0.75), whereas it was longer in 129 mice compared to B6 and D2 mice (strain difference *F*_2,130_ = 9.68, *P* = 0.0001).

#### 3.1.5. Marble burying and nest building

**Figure 5:**
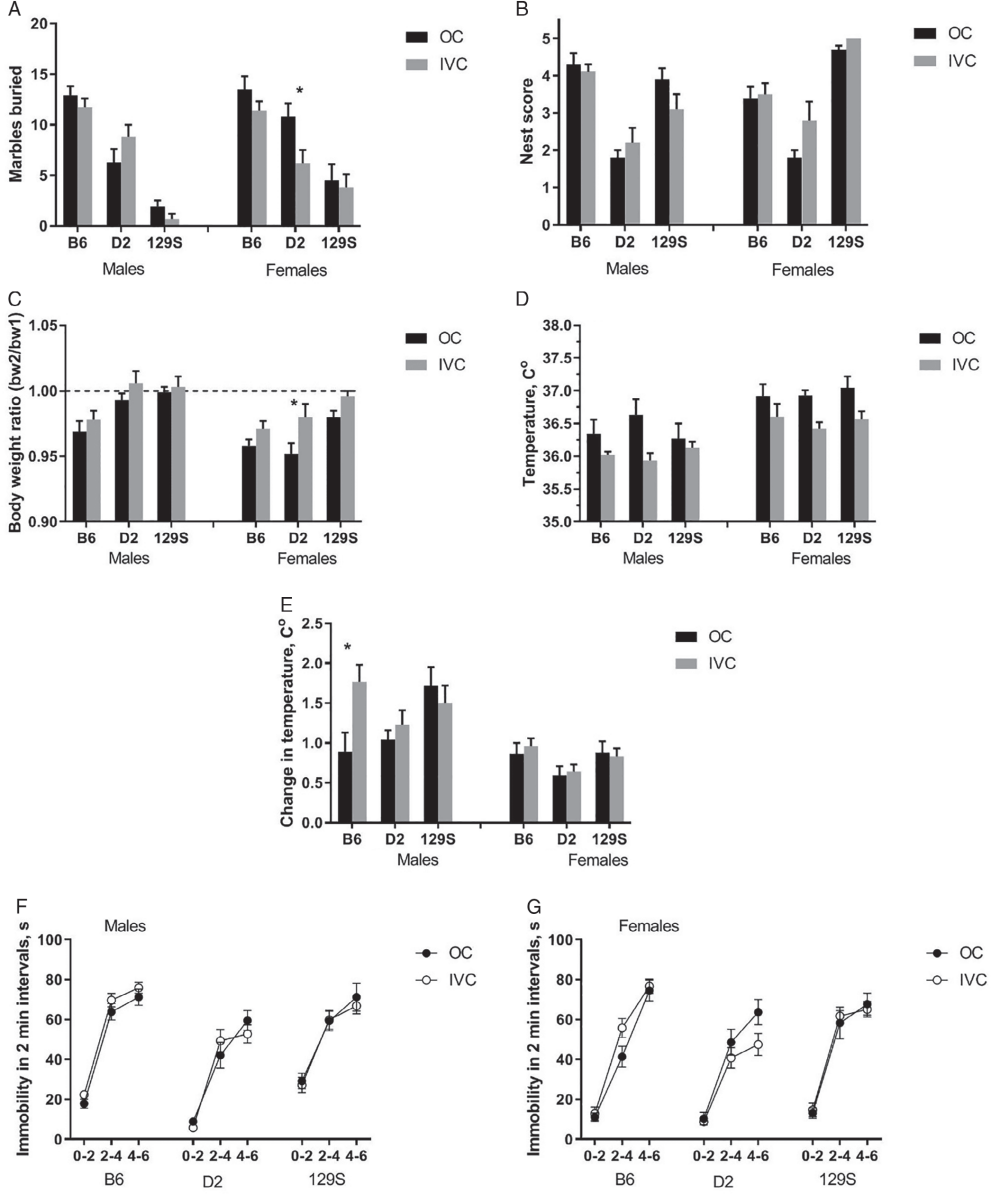
Species‐specific and stress‐related measures. A, Marble burying test—number of marbles completely hidden after 30 minutes. B, Nest construction—quality of the nest assessed in 5‐point scale. C, Change in body weight after single housing for one night—ratio between the second and initial measurements. D, Basal rectal temperature. E, Stress‐induced hyperthermia (difference between two measurements of rectal temperature in 10‐minute interval). F, Forced swim test—immobility in 2‐minute intervals, male mice. G, Forced swim test—immobility in 2‐minute intervals, female mice. Data are expressed as mean ± SEM; N = 10 B6 males in open field (OC); N = 12 for all other groups; *P < 0.05 Bonferroni post‐hoc comparison

Burying activity (number of marbles completely covered by bedding material after 30 minutes, Figure 5A) was not affected by housing conditions (*F*_1,130_ = 3.33, *P* = 0.07), and although significant strain difference was found (*F*_2,130_ = 70.81, *P* < 0.0001, B6 > D2 > 129), there was no interaction between environment and strain (*F*_2,130_ = 0.12, *P* = 0.89).

Nest construction (Figure 5B) was not affected by housing conditions (*F*_1,130_ = 0.80, *P* = 0.37). Significant strain difference was established (*F*_2,130_ = 55.04, *P* < 0.0001, B6 = 129 > D2) without interaction between environment and strain (*F*_2,130_ = 2.63, *P* = 0.08). Interestingly, we found that after one night of individual housing (needed for assessment of nest building and measuring SIH), the decrease in body weight (Figure 5C) was affected by housing conditions (*F*_1,130_ = 11.72, *P* = 0.0008, larger in OCs compared to IVC), by strain (*F*_2,130_ = 13.30, *P* < 0.0001, more in B6 than D2 and 129) and sex (*F*_1,130_ = 21.76, *P* < 0.0001, more pronounced in females than in males).

#### 3.1.6. Stress‐induced hyperthermia

Basal rectal temperature (Figure 5D) was significantly reduced in IVC animals (*F*_1,130_ = 19.25, *P* < 0.0001). There was no strain difference (*F*_2,130_ = 0.05, *P* = 0.95), but basal temperature in females was significantly higher than in males (*F*_1,130_ = 31.86, *P* < 0.0001). No interaction between environment and strain or sex was showed. SIH (Figure 5E) was not affected by housing condition (*F*_1,130_ = 2.86, *P* = 0.09), although interaction between environment and strain was significant (*F*_2,130_ = 3.55, *P* = 0.03). Moreover, female mice displayed reduced response as compared to males (*F*_1,130_ = 35.66, *P* < 0.0001) whereas interaction between environment and sex was not significant (*F*_1,130_ = 1.7, *P* = 0.19).

#### 3.1.7. Forced swimming test

Immobility time (Figure 5F,G) in this acute stress model was not affected by housing conditions (*F*_1,130_ = 0.004, *P* = 0.95) whereas the strains differed significantly (*F*_2,130_ = 1.92, *P* < 0.0001, B6 = 129 > D2) and females displayed less immobility than males (*F*_1,130_ = 5.0, *P* = 0.03).

## 4 DISCUSSION

With the present study we wanted to address in a systematic manner the possible modifying effect of IVC housing on mouse behavioral phenotype. In order to do so, we tested the male and female mice of three common inbred strains after housing them for 6 weeks (starting from the age of 4 weeks) in the IVC or OCs.

There is no doubt that the housing conditions have a robust influence on the behavior and physiology of laboratory mice.^11^ However, the observed effects can depend on the sex and strain of the animals on the one hand, and on the cage and laboratory environment on the other hand.^24^, ^25^, ^26^ For the latter part, the major difference can arise from the housing system used in the animal facilities—either IVC or, nowadays explicitly on a regressive basis, conventional OCs.^27^, ^28^, ^29^

A major variable between the different housing conditions is a microenvironment and ventilation affecting on it. In the IVC, forced ventilation has an effect on ammonia and carbon dioxide concentrations, relative humidity and temperature because of the differences in ventilation rate.^14^, ^30^ The IVC seem to keep the ammonia level in a more tolerable concentration compared to OCs.^14^ Being exposed to a reduced oxygen concentration may induce hypoxia causing impairments in animal physiology.^31^ Mice in the IVC can be disturbed by high ventilation rates,^32^ suffer from noise and vibration originating from the ventilation system^33^ and limited climbing possibilities.^34^ Acoustic environment, which differs between the housing conditions, may have a different effect on mouse strains with different hearing sensitivity.^35^

It has been suggested that ventilation, which varies among caging systems, has an effect on mice growth and behavior.^36^ In the present study, no difference in weight gain was detected because of housing conditions, whereas controversial results have been reported by others. Significant differences in body weight development have been reported between different IVC systems^14^ and after changing the housing system from OC to the IVC.^37^ On the other hand, the housing system did not have any impact on growth in male rats.^38^ The diet may have a direct influence on the growth, and there is a growing and compelling evidence on the role of gut microbiota on brain and behavior.^39^ In our study, the mice were housed in the specific pathogen free environment (both IVC and OCs) and fed with sterilized food. However, traditional OCs in many laboratories have been maintained in conventional rooms where the animals are fed with nonsterilized food. It would be interesting and important to investigate, how these different factors interact (eg, feeding mice in the OCs with sterilized or nonsterilized food).

Previous studies regarding the housing conditions or related topics have been conducted with multiple different types of cages, racks and ventilation control units. For instance, the traditional OCs can be truly open (ie, covered only with wire lids) or have a filter top attached to reduce pollution. These cages might be stored in ventilated cabinets or in room air. In addition, the size of the cages varies. An important difference between housing conditions may arise actually from the known fact that size of the cage and animal density has an influence on agonistic behavior.^14^ Individually ventilated cages and racks are available from a number of manufacturers: for example, BioZone (VentiRack) with Makrolon type II cages,^14^, ^15^, ^40^ Tecniplast with SealSafe cages,^14^, ^15^, ^26^, ^37^, ^41^, ^42^ Thoren Caging Systems Inc. (ie, Maxi‐Miser),^30^, ^43^, ^44^, ^45^ Alternative Design Manufacturing and Design (Max 75),^41^ Innovive (Innorack),^46^ Allentown^25^, ^26^, ^47^ and AirLaw Pty Ltd.^48^, ^49^ Among the other characteristics, the major difference between the systems can be driven by positive or negative pressure used for forced air exchange. Therefore, enormous variation exists between conditions, micro‐ and macro‐environments used in different facilities. This makes any attempts to make simple comparisons based on existing literature just between “IVC” and “open” cages very difficult, because of wide variety of details which can have an effect on the outcome.

For examination of the behavioral profile after being housed in the IVC or OCs, the mice in our study were subjected to a comprehensive test‐battery, including anxiety‐like behavior in the light‐dark box, exploratory activity in open field, testing of social approach to unfamiliar conspecific, marble burrowing and nest building as species‐specific behaviors, SIH and forced swimming test. It is important to note that duration of housing in the IVC before the experiments is another variable which may affect the variable results between the studies (Table 1). Usually, the animals at commercial breeding centers are born in OCs (personal communication), and after delivery to the research institution may experience different duration of housing in new environment. In general, 10‐14 days is suggested for acclimatization.^50^ However, adaptation after changing from the OC to IVC environment might require even more time. Moreover, the developmental stage of the animals during transfer (adolescent or adult) can influence the adaptation. Although we did not find any systematic study where the behavior and welfare of the mice after transportation at different ages has been investigated, there is evidence that rats display long‐lasting changes in physiological and behavioral parameters after transportation^51^ and these effects can be sex‐dependent.^50^ Moreover, there is a large variation in the type and duration of housing environment before the start of behavioral testing (Table 1). In fact, we did not find any study where comparison of the animals with life‐long history (ie, born and maintained throughout duration of the study) in the respective housing conditions (IVC or open) was compared. Therefore, the timing of changes in the housing environment (moving from IVC to open or vice versa) and the time allowed for adaptation after this move may be critical parameters which need to be considered in the design of experiments.

For the purpose of wider applicability we tested the male and female mice of three inbred strains (C57BL/6J, DBA/2J, 129S2/Sv). The differences in the behavioral characteristics of these mouse strains are well established.^19^, ^52^, ^53^, ^54^ We focused on the emotional (anxiety‐like, exploratory, stress‐related), social and species‐specific aspects of behavior, as we hypothesized that these areas may be the most influenced by housing in the IVC because of the possible deprivation of sensory information (eg, odors, vocalizations). The LD box and open‐field arena are commonly used for the assessment of exploratory and anxiety‐like behavior in rodents. In both tests, the C57BL/6 scored as the most active and explorative strain, whereas 129S2/Sv mice displayed low activity and high anxiety‐like behavior and the DBA/2 mice were in between of these two extremes. This ranking is well in line with the previous studies.^52^ General locomotor activity (total distance moved during the test) was not different between two housing conditions in any of the tests. This finding is in agreement with some previously published data where the housing system did not have an effect on the locomotor activity in novel arenas,^25^, ^42^ whereas the others have shown either increased^49^ or decreased^34^ locomotor activity of mice housed in the IVC. However, in our study the IVC‐housed mice displayed enhanced avoidance (expressed as a percentage of distance and time) of the light compartment in the light‐dark box, suggesting increased anxiety‐like behavior. In contrast, testing in the OF did not show any clear‐cut difference between two housing conditions, except for the time spent in the center which was slightly increased in the IVC group, pointing to possible reduction in anxiety‐like behavior (opposite to the light‐dark test). However, it is important to note that the latter finding was affected by genetic background ‐ the B6 mice from OCs displayed reduced activity in the center, whereas the D2 mice from OCs showed increased activity. The discrepancy between the results of the LD box and OF (and in general, between different “unconditioned,” “exploratory” tests for anxiety‐like behavior) may be because of differences in the strength and type of aversive stimulation (bright light, openness, height etc.).^55^

We did not see any significant difference in expression of directed exploration towards conspecific (a same‐sex, unfamiliar mouse) during the social approach test, whereas others have shown that history of housing in the IVC increased social activity.^48^ The observed strain differences in activity and social preference were largely corresponding to the earlier comparisons.^56^ Neither marble burying behavior nor nest construction was influenced by housing condition. The differences between the strains for these parameters were consistent with the previous reports.^57^, ^58^ For testing the nest building, the mice were single‐housed overnight in a clean cage with food and water freely available. Single‐housing can be viewed as a stressor, although the effects of this kind of manipulation have been usually monitored after several days or even weeks. One result of the acute stress can be a loss of body weight.^59^ Interestingly, we found that overnight weight loss was more severe in the OCs. Another unexpected outcome was a higher basal temperature measured in mice housed in the OCs. Based on these two findings it could be speculated that acute single‐housing is more stressful in OCs than in the IVC's, although further experiments are needed for validation of these effects. Nevertheless, the SIH was not different between two housing conditions. As a last procedure in the battery, we conducted the forced swim test for measuring the coping in a situation of acute stress. It appeared, that housing condition did not have any effect on the immobility time (“behavioral despair”). However, the strain ranking (D2 mice floating less than B6 or 129) was in line with the earlier findings.^19^, ^53^, ^60^

In summary, we have characterized some basic behavioral phenotypes in three commonly used inbred mouse strains after long‐term housing in individually ventilated cages and compared it to housing in OCs, otherwise maintained in the same environment. It can be concluded that under these conditions the IVC housing did not change the well‐known differences between the strains. Therefore, the external validity of research findings is most likely not compromised by this change in housing conditions. However, within the strains the differences may occur, dependent on the specific tasks applied. Therefore a critical re‐evaluation of the phenotypes in genetically modified mouse strains is warranted. In future studies, the caging system used needs to be explicitly mentioned in the methods.
